# Patterns of selection in the evolution of a transposable element

**DOI:** 10.1093/g3journal/jkac056

**Published:** 2022-03-09

**Authors:** Julie Dazenière, Alexandros Bousios, Adam Eyre-Walker

**Affiliations:** School of Life Sciences, University of Sussex, Falmer, Brighton BN1 9RH, UK

**Keywords:** transposable elements, adaptive evolution, purifying selection, plants, maize

## Abstract

Transposable elements are a major component of most eukaryotic genomes. Here, we present a new approach which allows us to study patterns of natural selection in the evolution of transposable elements over short time scales. The method uses the alignment of all elements with intact *gag/pol* genes of a transposable element family from a single genome. We predict that the ratio of nonsynonymous to synonymous variants in the alignment should decrease as a function of the frequency of the variants, because elements with nonsynonymous variants that reduce transposition will have fewer progeny. We apply our method to Sirevirus long-terminal repeat retrotransposons that are abundant in maize and other plant species and show that nonsynonymous to synonymous variants declines as variant frequency increases, indicating that negative selection is acting strongly on the Sirevirus genome. The asymptotic value of nonsynonymous to synonymous variants suggests that at least 85% of all nonsynonymous mutations in the transposable element reduce transposition. Crucially, these patterns in nonsynonymous to synonymous variants are only predicted to occur if the gene products from a particular transposable element insertion preferentially promote the transposition of the same insertion. Overall, by using large numbers of intact elements, this study sheds new light on the selective processes that act on transposable elements.

## Introduction

Transposable elements (TEs) are DNA sequences that can duplicate themselves and relocate from 1 chromosomal locus to another. They are divided into 2 main classes; class I elements [long-terminal repeat (LTR) and non-LTR retrotransposons] spread via a copy-and-paste pathway that involves an RNA intermediate, whereas class II elements (DNA transposons) transpose via a cut-and-paste pathway; both can result in a net increase of the TE copy number (Wicker *et al.* 2007; Sultana *et al.* 2017). TEs typically transmit vertically within hosts through the germline, but increasing evidence suggests that horizontal transfer of TEs can occur between species (Gilbert and Feschotte 2018; Zhang *et al.* 2020). Due to their activity over evolutionary time, TEs account for ∼50% of most primate genomes (Lee *et al.* 2015) and up to 80–90% of the genome of some plants [Jiao *et al.* 2017; International Wheat Genome Sequencing Consortium (IWGSC) *et al.* 2018]. As such, TE activity is a major determinant of DNA sequence diversity and a key driver of species evolution (Lisch 2013; Chuong *et al.* 2017).

TEs can be potentially harmful because they can integrate into genes and disrupt their function (Hancks and Kazazian 2016; Qian *et al.* 2017). They can also insert into promoters and regulatory sequences in the vicinity of genes, which can reduce expression levels by attracting silencing mechanisms and increasing local DNA methylation levels (Hollister and Gaut 2009). TEs can also have negative consequences because they generate ectopic recombination (Petrov *et al.* 2011; Bourgeois and Boissinot 2019), impose an energetic cost on genomes (Bousios and Gaut 2016), and trigger intragenomic conflict when they capture fragments of host genes (Muyle *et al.* 2021). Generally, TEs are thought to reduce host fitness and they have even been implicated with various diseases such as cancer in humans (Hancks and Kazazian 2016; Burns 2017). However, TEs can potentially be advantageous for many of the same reasons, for example by providing exons or introducing promoter and enhancer elements near genes (Bourque *et al.* 2018). In plants, there are several well-documented cases of agriculturally important traits that are caused by TE insertions (Butelli *et al.* 2012; Hou *et al.* 2012; Lisch 2013; Makarevitch *et al.* 2015).

TEs evolve in 2 separate dimensions. The first is through amplification within the host genome. After a new TE insertion occurs, it is polymorphic in the host population—some individuals have the element at this chromosomal position and others do not. This mutation, like all mutations, is subject to population processes of genetic drift and natural selection (Le Rouzic *et al.* 2007; Quadrana *et al.* 2016; Bourgeois and Boissinot 2019; Baduel *et al.* 2021). The vast majority of these insertions will be lost from the population either because of genetic drift, or selection against them, if they are deleterious. A few insertions may also spread through the population, again either because of drift or selection, if the insertion is advantageous.

TEs also evolve in another dimension; they themselves evolve. This aspect of the evolutionary process has not been as extensively studied (Boissinot and Furano 2001; Costas 2001; Belshaw *et al.* 2004; Baucom *et al.* 2009; Ma *et al.* 2019; Zhang *et al.* 2020). It has been shown that the evolution of retrotransposons is largely dominated by negative selection both between and within families (Boissinot and Furano 2001; Costas 2001; Belshaw *et al.* 2004; Baucom *et al.* 2009; Ma *et al.* 2019; Zhang *et al.* 2020). Occasionally, positive adaptive evolution has been detected, as in the coiled coil region of ORFI of the human L1 LINE element (Boissinot and Furano 2001). In contrast, there seems to be little evidence of selection acting on DNA transposons, except when these TEs are transferred between species (Zhang *et al.* 2020).

When a new TE insertion occurs, it will start to accumulate mutations. These may be neutral with respect to transposition if the element inserts into a region of the genome from which it cannot further transpose, or if the changes have little effect on the probability of transposition; for example, if the mutations are synonymous. However, many mutations in the TE sequence will reduce the rate of transposition. As a consequence, as most TE insertions age so they will have fewer and fewer progeny; TEs are in a race to generate new copies of themselves before their sequence degenerates so that they can no longer transpose. All the TEs from a particular family in a single genome or in a population are connected to each other by a phylogeny. A consequence of the accumulation of mutations, which reduce transposition, is that internal branches in the tree should have fewer of these mutations, because internal branches represent elements that have successfully transposed (ignoring duplication of the locus), and internal branches with more daughter branches represent more successful elements. We can detect this pattern by considering the ratio of nonsynonymous (vN) to synonymous (vS) variants, assuming that most synonymous mutations have no effect on transposition. We refer to mutations in the phylogeny as variants since they are neither substitutions nor polymorphisms; i.e. there is no guarantee that they are fixed in the species, as we would expect for a substitution, and although they are quite likely to be polymorphic within the population, because the element that the variant appears in is probably polymorphic in the population, the variant is defined relative to other copies of the element, and so referring to them as polymorphisms is inappropriate. We thus predict that branches internal to the tree should have lower vN/vS than external branches and that vN/vS should decline as a function of depth in the tree (i.e. branches with 3 descendant branches should have lower vN/vS on average than those with 2). Occasionally a mutation might arise that increases the rate of transposition. Such an increase has 2 effects; it increases the number of elements being produced, but at the same time it can reduce the fitness of the host. It has been shown that an increase in transposition can be favored even if it is deleterious to the host (Charlesworth and Charlesworth 1983; Burt and Trivers 2006). If this is the case then the net effect will be to increase the number of progeny elements and the branch will have a high value of vN/vS.

An important caveat to these predictions for vN/vS is that the gene products of a TE insertion act in *cis* to generate copies of that particular locus, not copies of other loci of the same TE family in the genome. If the gene products from 1 insertion help in *trans* other TE loci transpose, then even those TE copies with mutations that would render them otherwise incapable of transposition, will transpose and hence their vN/vS will be ∼1 (Belshaw *et al.* 2004; Zhang *et al.* 2020).

It is well established that many TE copies contain debilitating mutations, such as stop codons and frameshift mutations in their coding sequences. What has rarely been demonstrated is the slow death of many TEs, through the accumulation of mutations that reduce transposition, and how those elements that avoid these, keep the lineage alive. In the only analysis of this kind, Belshaw et al. (2004) showed that vN/vS was lower for the internal than external branches for human endogenous retroviruses.

We predict that vN/vS should typically be lower for internal than external branches. However, a challenge in the analysis of many TE families is their size and the speed at which they have expanded; inferring a robust phylogeny can therefore be difficult. We therefore developed a new method in which we align all the TE sequences from a single genome, and consider the variation in this alignment; in this alignment, we assume that a variant present in a single TE sequence appeared on a terminal branch of the tree (or it appeared on an internal branch and there was a back-mutation), 1 that is present in 2 copies occurred on a branch ancestral to 2 of the TEs in the genome and so forth. Hence, we can infer the position at which the mutation occurred in the tree from its frequency. We therefore have 2 predictions. First, if synonymous mutations are neutral and nonsynonymous mutations are neutral or deleterious in terms of TE transposition, then vN/vS should decline as a function of the frequency of the variant in the alignment. Second, if some mutations are advantageous to the TE, then this should lead to an increase in vN/vS amongst the highest frequency variants. Note that in contrast to an advantageous mutation spreading through a population, in which the advantageous allele can rapidly spread through the whole population, an advantageous mutation never spreads immediately through all the TE copies in a single genome; the new variant can proliferate but there still remain all the elements that were already integrated into the genome. We test these predictions on a lineage of LTR retrotransposons that are found in plants. We focus only on the subset of elements that are potentially capable for autonomous transposition based on the completeness of their coding domain. We find clear evidence of negative selection, but no evidence of positive selection.

## Materials and methods

### Identification of intact Sirevirus elements

We ran MASiVE (Darzentas *et al.* 2010) to identify full-length Sirevirus elements in the genomes of the 7 species used in this study. Supplementary Table 1 contains information on the genome versions, source links and citations for these species. Similar to other de novo LTR retrotransposon identification algorithms, MASiVE identifies full-length Sireviruses based on the presence of structural features (e.g. LTRs, primer binding site, polypurine tract, target-site duplication) and positive hits with the core domain of the *reverse transcriptase* and *integrase* genes using the Pfam Hidden Markov Models (HMM) PF07727.9 and PF00665.21. However, it is not guaranteed that these elements are intact in terms of their coding domains and if they are competent for autonomous transposition (Supplementary Fig. 1); in fact, it is likely that most elements have acquired 1 or more mutations after integration in the genome that disrupted the *gag* and *pol* ORFs. Generally, the proportion of TEs within a family that are intact elements is unknown and identifying them requires substantial resources and TE expertise.

For this study it was necessary to characterize these elements and, hence, we devised the following pipeline: For every species, we first produced a multiple alignment using Mafft G-INS-i algorithm (Katoh and Standley 2013) of all Sirevirus elements based on the HMM domain of the *reverse transcriptase* gene. Due to the high numbers of elements in maize (Table 2), we used the CD-HIT clustering package (Li and Godzik 2006) to reduce the number of elements prior to the alignment. We required a 95% identity threshold (–c 0.95) and a coverage of at least 90% for every sequence pair (-aL and -aS both at 0.9). Every sequence was placed in the most similar cluster and not the first one that met the thresholds (–g 1). We then ran FastTree (Price *et al.* 2009) to generate maximum likelihood phylogenetic trees, which were visualized using FigTree (http://tree.bio.ed.ac.uk/software/figtree/, version 1.4.3; accessed 2021 June 30). We assigned elements into families based on the branching pattern and bootstrap support. The addition of known Sirevirus exemplars in maize from Bousios *et al.* (2010) and the maize TE database was used to assign branches to specific family names. We then ran getorf from the EMBOSS suite (Rice *et al.* 2000) with -minsize 1,000 to identify long open reading frames (ORFs) within the internal domain (i.e. excluding the LTRs) of each element and hmmscan from the HMMER software (hmmer.org; accessed 2021 May 13) using a list of known HMMs for LTR retrotransposons (Supplementary Table 2) to annotate the ORFs as part of the *gag* or *pol* polyprotein. The length and start positions of the *gag* and *pol* ORFs were then plotted, while for *pol* we additionally required for the presence of the amino acid motif ADIFTK that is conserved among *Copia* LTR retrotransposons (Pearce *et al.* 1999). The motif lies a short distance upstream of the 3′ end of the *pol* gene and was therefore used as an anchor to only keep *pol* ORFs that were complete on the 3′ end. An example of this process is shown in Supplementary Fig. 2.

**Table 2.** Plant species and Sirevirus families were included in this study.

Species	Family	Full-length elements	Intact Sireviruses
*Zea mays*	*Opie*	10,788	2,445
	*Ji*	10,563	2,345
	*Hopie*	374	140
	*Giepum*	504	139
	*Jienv*	279	40
*Asparagus officinalis*	Family 1	92	22
	Family 2	457	58
*Glycine max*	Family 1	403	64
	Family 2	842	404
*Helianthus annuus*	Family 1	1,360	60
*Musa acuminata*	Family 1	263	134
*Panicum hallii var. hallii*	Family 1	31	13
	Family 2	71	7
	Family 3	56	12
	Family 4	243	44
*Sorghum bicolor*	Family 1	70	11
	Family 2	62	5
	Family 3	200	33
	Family 4	213	32

In maize, known TE exemplars were used to assign each element to a known family (see *Materials and Methods*). Simple names (e.g. Family 1, Family 2) were used for species with no exemplars.

Finally, the junctions of the 4 genes within *pol* were identified as follows: *protease* was from the beginning of *pol* up till the beginning of the GAG-pre-integrase Pfam domain (PF13976), which was hence defined as the *protease*/*integrase* junction. This matches the boundaries of these 2 genes as identified by Peterson-Burch and Voytas (2002). The C-terminus of the *integrase* is generally poorly conserved across *Copia* elements, but Sireviruses contain the ILGD motif a short distance (10–20 amino-acids) upstream of the 3′ end of the gene (Peterson-Burch and Voytas 2002). In maize Sireviruses, this motif is followed after ∼50 amino acids by the beginning of the *reverse transcriptase* Pfam domain (PF07727). We therefore approximately set the *integrase*/*reverse transcriptase* junction to be 30 amino acids upstream of PF07727. The junction of the *reverse transcriptase* with the *ribonuclease* is also not precisely defined in the literature. However, *ribonuclease* starts with a highly conserved D_10_E_48_D_70_ motif (Malik and Eickbush 2001), and the region of the first aspartic acid can be readily identified in Sireviruses. The aspartic acid also lies ∼85 amino-acids downstream of the *reverse transcriptase* Pfam domain (PF07727) and it does not overlap the last conserved domain of the *reverse transcriptase* gene as it was identified by Xiong and Eickbush (1990). We approximately set the *reverse transcriptase* with the *ribonuclease* junction to be 15 amino acids upstream of the first aspartic acid of the D_10_E_48_D_70_ motif.

### Multiple sequences alignments

Our method relies on the number of vN/vS identified in a multiple sequence alignment (MSA). The TE sequences in each family are moderately divergent from each other and they contain a number of indel mutations, so aligning them was challenging. We tried several approaches; MAFFT (Katoh and Standley 2013) and MACSE (Ranwez *et al.* 2018) both introduced substantial numbers of gaps; in addition, in the case of MAFFT, they were not multiples of 3. We therefore used TranslatorX (Abascal *et al.* 2010) in association with MUSCLE (Edgar 2004) to align the sequences at the amino acid level. Visual inspection suggested that these alignments were reasonable; i.e. by aligning at the amino acid level we do not allow indels that introduce a frameshift, but frameshifts are apparent as sections of the alignment which align poorly in the sequences that are genuinely frameshifted. We found only 1 such case in the *gag* gene of the *Opie* family. Such alignment problems will introduce noise into our analysis, by generating vN/vS at the same frequency.

Our alignments contain multiple gaps in certain regions. To investigate whether the quality of our alignments affected our results we repeated our analysis. First, as our intact elements slightly vary in length, we chose only those sequences within a selected range (Supplementary Fig. 2) whose length class contained at least 10 sequences. We then realigned the sequences. Second, we edited the original alignment to remove those sections that had multiple gaps. Our results remained qualitatively unchanged, so we proceeded with the original alignments.

### Determination of the vN/vS ratio

We want to estimate the rate at which vN/vS accumulate in the TE sequences. One option would be to construct the phylogeny of the TE sequences and then estimate the rate at which variants accumulate using one of the many methods which have been developed to estimate rates of synonymous and nonsynonymous substitution. However, the phylogeny is poorly resolved for our TE families since they are relatively young. We therefore developed a simple counting method in which synonymous and nonsynonymous mutations were equally likely to appear and be counted. To do this we focused on groups of codons, generally 4 codons. For example, the 4 codons from Phenylalanine and Leucine—TTT, TTC, CTT, and CTC. Here the synonymous and nonsynonymous mutations involve the same mutation C<>T, and hence are expected to have the same mutation rate (ignoring context effects) and we can score both vN and vS even if they occur together (Table 1). We had 5 sets of 4 codons in which the synonymous and nonsynonymous mutations were the same, and 3 sets of 4 codons in which the synonymous and nonsynonymous mutations were the complement of each other; for example, the Isoleucine and Valine codons ATT, ATC, GTT, and GTC; here the synonymous mutation is T<>C and the nonsynonymous mutation is its compliment, A<>G. We also included one set of 16 codons, the codons of Proline, Threonine, Alanine, and 4-fold degenerate codons of Serine (i.e. all codons of the form NCN). Here synonymous and nonsynonymous mutations are expected to occur at equal rates (assuming context effects are minimal); for example, a TCT codon is equally likely to give rise to TCA and ACT, representing synonymous and nonsynonymous changes. The list of codons is given in Supplementary Table 3. In some cases, the group of 16 codons could give rise to tri-allelic sites. In these cases, we took the frequency of the rarest allele. All of these sets are independent of each other—they do not share any codons in common. There are other sets that we could use but unfortunately these are not independent. For a codon site to be included in the analysis it had to contain at least 10 instances of a set of codons (see codon 4 in Table 1). However, one codon site could contribute to multiple codon sets (see codon 5 in Table 1). In terms of the frequency of the variant we always consider the minor allele, and the frequency is considered across the whole alignment, not just the set of codons in which the variant appears. For example, codon 5 in Table 1 has a synonymous variant at a frequency of 1 in 10; let us imagine that there are 100 sequences; the variant frequency would then be 1 in 100.

**Table 1.** Examples of how synonymous and nonsynonymous variants are counted.

Sequence	Codon 1	Codon 2	Codon 3	Codon 4	Codon 5
1	TTT	TTT	TTT	TTT	TTT
2	TTT	TTT	TTC	TTC	TTC
3	TTC	CTT	CTT	AGA	CTT
…	…	…	…	…	…
10	TTC	CTT	CTT	AGG	CTT
11	TTC	CTT	CTT	GCT	AAA
12	TTC	CTT	CTT	ACT	AAA
…	…	…	…	…	
20	TTC	CTT	CTT	TTT	AAG
Synonymous variant count	1	0	1	0	2
Nonsynonymous variant count	0	1	1	0	1
Notes				No codon set has 10 instances	Multiple codon sets included

### Sampling of *Opie* and *Ji*

The sampling of the 2 biggest families of maize, *Opie* and *Ji*, was performed on the dataset of intact elements. For each of these 2 families, we sampled of 130 sequences at random. The pipeline was then applied as previously described.

## Results

### TE datasets and plant species used in this study

We are interested in whether we can detect the signature of natural selection acting upon the sequence of TEs. We chose to investigate this among Sirevirus LTR retrotransposons, a TE lineage that is specific to plants (Bousios and Darzentas 2013) and often occupies a substantial proportion of the genome of their hosts (Bousios *et al.* 2012b). In the maize B73 genome, for example, there are 5 distinct Sirevirus families that collectively occupy ∼21% of the 2,300Mb genome (Bousios *et al.* 2012a). Among the 5 families, *Opie* and *Ji* have been very successful with each representing ∼10% of the genome (Bousios *et al.* 2012a) and with 10,778 and 10,563 full-length elements respectively (Table 2). In contrast, *Giepum*, *Hopie*, and *Jienv* are found in much lower copy numbers (Table 2). These elements are considered full-length, because they contain all the structural features of complete LTR retrotransposons that are used by the various *de novo* TE identification pipelines: i.e. the presence of LTRs, a primer binding site, a polypurine tract, target-site duplication, and the core domains of the *reverse transcriptase* or *integrase* genes (Supplementary Fig. 1a). However, not all these elements are potentially functional due to mutations that can interrupt their genes. We consider that there is little point in testing for natural selection in elements that are clearly inactive based on their coding potential. We therefore identified a subset of elements that contain uninterrupted *gag* and *pol* open reading frames (see *Materials andMethods*) and refer to them as “intact” (Supplementary Fig. 1b). For *pol*, we further identified the junctions between *protease*, *integrase*, *reverse transcriptase*, and *ribonuclease* so we could analyze them separately. In maize, this approach identified 2,445, 2,345, 140, 139, and 40 intact elements for *Opie*, *Ji*, *Hopie*, *Giepum*, and *Jienv* respectively (Table 2). Because *Ji* and *Opie* are so much more numerous than *Hopie*, *Giepum*, and *Jienv*, we randomly chose 130 sequences from *Opie* and *Ji* so as to have comparable population sizes across families for further analysis, and to avoid the problem of multiple hits at sites, which can inflate vN/vS in the low frequency categories. Besides maize, we also identified full-length Sireviruses in a collection of monocot and eudicot species using the MASiVE annotation pipeline (Darzentas *et al.* 2010) and included in the analysis Sirevirus families that contained >5 intact elements (Table 2).

We aligned the genic sequences from each family in each species. Because we do not have a well-resolved phylogenetic tree for the elements in each family, and hence were unable to estimate rates of nonsynonymous and synonymous change along each branch, we counted the number of vN and vS in groups of codons in which vN and vS were equally likely to occur and be scored (see *Materials and* *Methods*).

### Patterns of selection at the family level

**Fig. 1.**
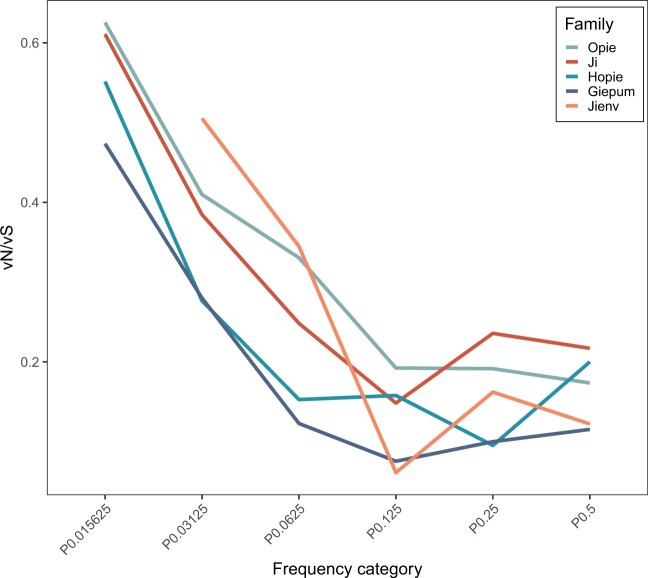
The value of vN/vS as a function of the frequency of the variants in the alignment for the 5 families of Sireviruses in maize. *Ji* and *Opie* are sampled to 130 sequences each, while *Giepum*, *Hopie*, and *Jienv* are the full datasets. P0.015625 refers to the frequency category 0 < x ≤ 2^−6^, P0.03125 to 2^−6^ < x ≤ 2^−5^…, and so on.

We begin our analysis by considering patterns of evolution in the maize families and for all genes combined. Because high-frequency variants are relatively rare, we combine frequency groups in the following scheme: we combine polymorphisms that have frequencies between 0 and 2^−6^, 2^−6^ and 2^−5^, 2^−5^ and 2^−4^ and so on until 2^−2^ and 2^−1^. In each family vN/vS is significantly less than one across all frequency categories (Supplementary Table 4), and it declines as a function of the frequency of the variants in the alignment across all 5 families before reaching an asymptote (Fig. 1; Table 3). There is therefore a clear signature of negative selection acting in each Sirevirus family against vS variants. The value of vN/vS does not vary significantly between families for most frequency categories (Table 4).

**Table 3.** Spearman correlation between vN/vS and variant frequency for each family and each gene.

Group		Spearman's correlation coefficient	*P*-value
Families	*Opie*	−1	0.003
	*Ji*	−0.829	0.058
	*Hopie*	−0.6	0.208
	*Giepum*	−0.771	0.072
	*Jienv*	−0.7	0.188
	Combined *P*-value		<0.001
Genes	*Gag*	−0.829	0.058
	*Integrase*	−0.771	0.103
	*Protease*	−0.771	0.072
	*Ribonuclease*	−0.714	0.111
	*Reverse transcriptase*	−0.943	0.005
	Combined *P*-value		<0.001

Note the correlation is calculated across frequency categories.

**Table 4.** Testing whether vN/vS differs between families and genes for each frequency category.

Analysis	Frequency category	Chi-square	*df*	*P*-value
Between genes	0 < x ≤ 1/64	12.09	4	0.02
	1/64 < x ≤ 1/32	8.69	4	0.07
	1/32 < x ≤ 1/16	10.65	4	0.03
	1/16 < x ≤ 1/8	15.2	4	0
	1/8 < x ≤ 1/4	9.89	4	0.04
	1/4 < x ≤ 1/2	11.09	4	0.03
	Total	67.61	24	<0.00001
Between families	0 < x ≤ 1/64	7.99	3	0.05
	1/64 < x ≤ 1/32	7.06	4	0.13
	1/32 < x ≤ 1/16	9.52	4	0.05
	1/16 < x ≤ 1/8	5.9	4	0.21
	1/8 < x ≤ 1/4	3.29	4	0.51
	1/4 < x ≤ 1/2	2.91	4	0.57
	Total	36.67	23	0.035

The chi-square value is given along with the degrees of freedom and the *P*-value. Note, in the family analysis there is only 3 df in the lowest frequency class because Jienv has only 40 intact elements and hence no variants in the lowest frequency class.

**Fig. 2.**
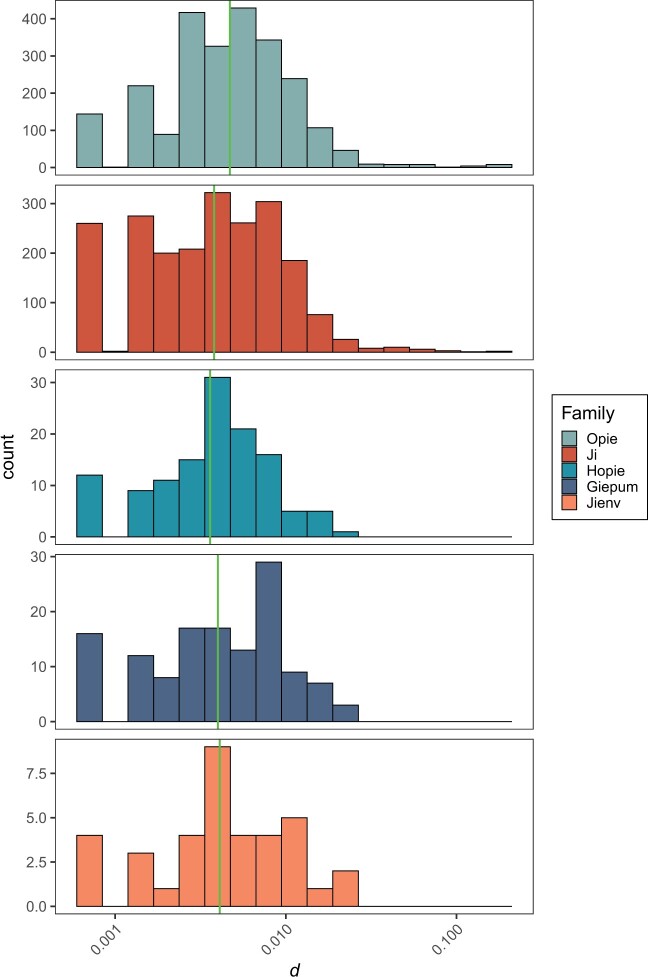
The distribution of relative ages across TE copies from each family. Histogram plots show the distribution of divergence (numbers of point mutations and indels per site) between the LTRs of each element. The green line represents the median within each family. Note that the *x*-axis is on a log10 scale.

The patterns of vN/vS are remarkably similar in the different families, even though 2 of the families are much more numerous than the others. A factor that might influence the patterns we observe is the age of the elements. We can potentially estimate the relative age of each element from the divergence between the 2 LTRs that flank each element; it is assumed that these are identical when the element first inserts and hence divergence between the LTRs can be used to estimate the relative age of each element; note that we do not attempt to estimate the absolute age, because the LTRs might have a function and hence evolve more slowly than the mutation rate. We find significant differences in the median relative age between families (Kruskal–Wallis test, *P* < 0.001) (Fig. 2), with pairwise Mann–Whitney tests suggesting that the median age of *Opie* elements is significantly different to *Ji* (*P* < 0.001), and to *Hopie* (*P* < 0.001). However, the differences in age are quite small.

### Patterns of selection acting on the TE genes

**Fig. 3.**
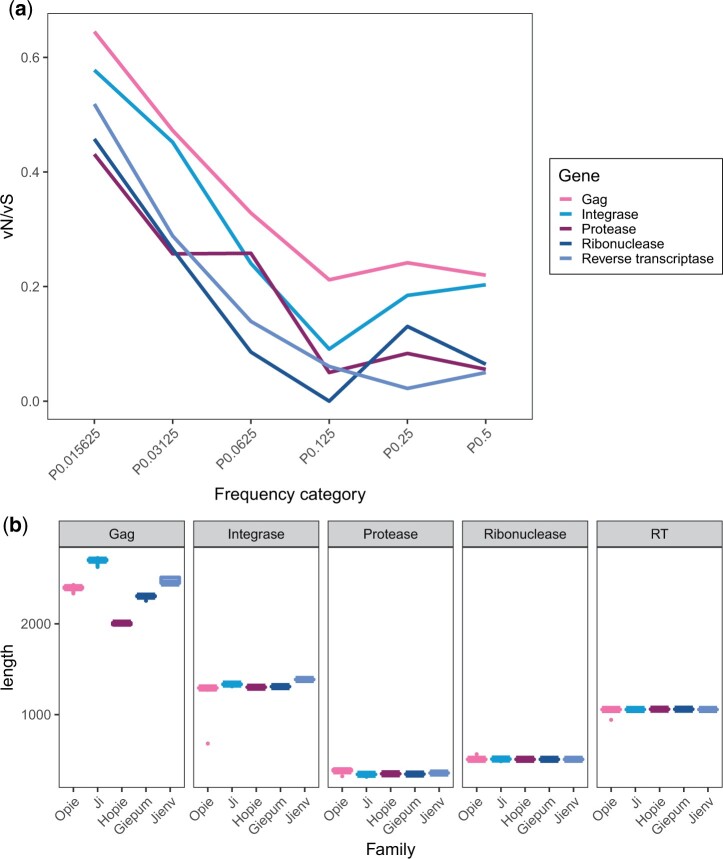
a) The value of vN/vS as a function of the frequency of the variants in the alignment for the 5 genes in the Sirevirus element, with the families combined. b) The length variation of the 5 maize families for each gene.

We now turn attention to whether there are differences in the pattern of natural selection between the genes in the Sirevirus sequence, by summing data across families for each gene. As we might have expected from the family analysis, we find that vN/vS declines significantly over the first 4 frequency categories before increasing in some of the genes, consistent with the action of adaptive evolution (Fig. 3a;Table 3). However, none of these increases are significant whether considered individually or collectively, as judged by chi-square tests comparing the sum of P0.25 and P0.5, against P0.125. The value of vN/vS varies significantly between the genes for all frequency categories (Table 4) with *integrase* and especially *gag* being less conserved than the other 3 genes. For *gag*, this is also reflected in the much higher length variation among families compared to the other genes (Fig. 3b). The average value of vN/vS over the last 3 frequency categories is 0.15 for the 5 genes.

### Patterns of selection in other plant hosts

**Fig. 4.**
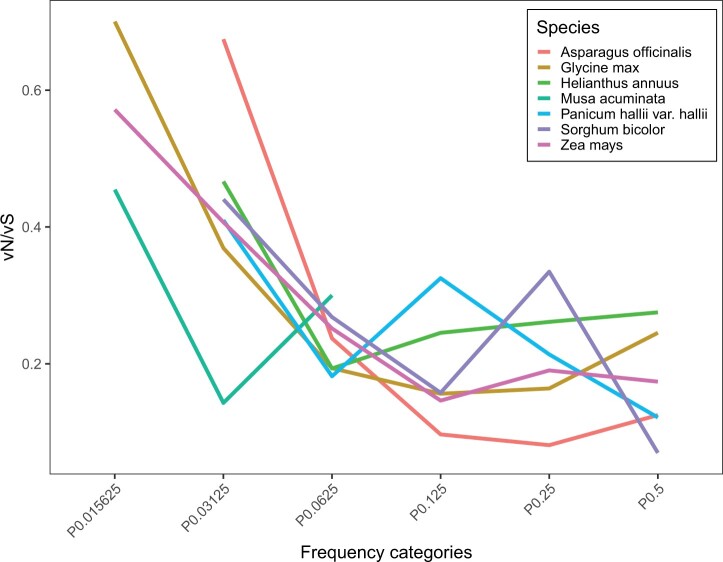
The value of vN/vS as a function of the frequency of the variants in the alignment for Sirevirus families in various plant species.

It is of interest to see if these general patterns are found in other species. We extracted Sireviruses from 6 other species which have between 31 and 1,360 full-length elements and between 5 and 398 intact elements (Table 2). As in maize we find that vN/vS declines across frequency classes before coming to an asymptote (Fig. 4).

## Discussion

We have investigated patterns of selection in intact Sirevirus elements within the genome of maize and other species using a new approach. We have aligned TE sequences from a single genome and considered the ratio of vN and vS as a function of the frequency of the variants in the alignment. We find that the ratio of vN and vS, vN/vS, declines as a function of the frequency of the variant in an alignment of the TE sequences in a single genome. This is expected; TE sequences that tend to accumulate deleterious vN variants are likely to be less able to transpose. The value of vN/vS across all genes for variants with frequencies in excess of 1/8 is 0.15 and this implies that at least 85% of nonsynonymous mutations in the Sirevirus sequence reduce transposition. The reason is as follows; let us assume that synonymous mutations are neutral (i.e. they have no effect on the rate of transposition), then the rate at which synonymous mutations accumulate is proportional to the mutation rate, *u*. In contrast, let us assume that nonsynonymous mutations are neutral or deleterious, in the sense that they reduce transposition; let the proportion that are neutral be *f* then the rate at which vS variants accumulate is proportional to *uf* and hence the ratio of vN/vS is simply an estimate of *f*; hence if vN/vS = *f *=* *0.15, this implies that 15% are neutral and 85% are deleterious. This is likely to be a lower estimate because some nonsynonymous mutations might increase the rate of transposition and some synonymous mutations might decrease the rate.

One of the challenges for any TE is avoiding parasitism. A functional and active TE will produce gene products that will allow it to generate new copies of itself that can be integrated into the host genome at new locations. However, these gene products can potentially be used by other elements to transpose themselves; a number of very successful TEs, such as SINEs in mammals and MITEs in plants, are incapable of transposing themselves, and duplicate by parasitizing the machinery of other autonomous TEs—LINEs and MLEs in the case of the SINEs (Weiner 2002) and MITEs (Feschotte *et al.* 2003) respectively. It is clearly in the interest of a TE to avoid this parasitism; the more gene products get diverted to other parasitic elements, the less likely the element that produced the gene products is to successfully transpose itself. The observation that vN/vS is very substantially less than one amongst the high-frequency variants suggests that each Sirevirus copy is successful at targeting most of its own gene products to their own transposition (Belshaw *et al.* 2004). The reason is as follows. If the gene products from a particular TE diffused throughout the cell, and helped other TE copies to transpose, then this would allow TEs to transpose that did not produce any useful gene product including those with substantial numbers of nonsynonymous mutations. Hence, vN/vS would be substantially higher and we would not observe vN/vS declining as a function of the frequency of the variants in the alignment. Our observation that vN/vS < 1 for higher frequency categories is evidence for this *cis*-targeting as Belshaw et al. (2004) first noted. This *cis*-targeting has been experimentally confirmed for LINE elements (Dombroski *et al.* 1991; Wei *et al.* 2001), but there is no evidence for this *cis*-targeting for the one LTR retrotransposon family that has been investigated in-depth, the Ty1 in yeast (Doh *et al.* 2014). In addition, the life cycle of an LTR retrotransposon makes it difficult to see how *cis*-targeting could be brought about (Burt and Trivers 2006). Zhang *et al.* have hypothesized that *cis* preference might arise if just 1 or 2 elements are transcribed in the germline, even if a given family has numerous copies in the genome (Zhang *et al.* 2020). This hypothesis requires further testing, but it is supported from preliminary evidence of a recent study on *Arabidopsis thaliana* and maize TEs (Panda and Slotkin 2020). In this study, the authors mapped long-read libraries of full-length mRNAs to TEs in an effort to pinpoint which copies of a family are truly expressed. This is not possible using short-read RNA-seq data due to the multimapping effect on TEs (Bousios *et al.* 2017). The authors found that only 4% of all annotated TEs in *A. thaliana* were expressed in a triple mutant that removes many layers of epigenetic silencing. In maize, they interestingly focused on *Opie*, and using libraries from different tissues they found that only 6 copies were expressed out of a total of ∼12,000. It is noteworthy that, similar to LTR retrotransposons, DNA transposons cannot perform *cis*-targeting because of their life cycle—the transposase is produced in the cytoplasm and diffuses back into the nucleus to cut and paste the element—and DNA transposons show vN/vS = 1 within a species (Zhang *et al.* 2020).

Our method has the potential to detect periods of adaptive evolution. If a TE undergoes a nonsynonymous mutation which allows the TE to transpose more often or which allows the TE to survive, then this TE will have more progeny, unless this increase in transposition imposes a significant cost on the host individual such that they have fewer offspring (Charlesworth and Charlesworth 1983; Burt and Trivers 2006). Such mutations are more likely to be nonsynonymous and hence we might expect to see an elevation in vN/vS amongst high-frequency variants. There are, however, 2 problems. First, negative selection is expected to lead to a decrease in vN/vS across frequency categories, so this may mask the signature of positive selection. Second, different advantageous mutations will have different effects; for example, one might lead to an increase in transposition such that 40% of the elements carry the advantageous mutation, whereas another might lead to only 10% of the TE population carrying the mutation; i.e. the signature of adaptive evolution is likely to be spread across many frequency categories. We observe vN/vS increasing for the higher frequencies, but this is not significant; unfortunately, we do not have enough high-frequency variants in our analysis.

The method makes a number of simplifying assumptions. We assume that the only manner in which a TE can make a copy of itself is through transposition, rather than through duplication of the genome, chromosome or part of the chromosome. We also assume that there is little or no gene conversion between TEs. Making these assumptions is unlikely to affect our results; both processes will tend to introduce noise in the analysis; i.e. we might have a TE which is incapable of transposition and which has accumulated equal numbers of nonsynonymous and synonymous mutations; all mutations should appear as singletons, unless there is duplication or gene conversion, which can potentially change a singleton into a 2 copies, hence elevating vN/vS in higher frequency categories.

It is conspicuous that the age distributions of the 3 smaller and the larger 2 Sirevirus families are remarkably similar. This is unexpected because one would expect these families to be transposing independently. What then could generate the similarity in the age profiles? There are 2 possibilities. Families of TEs are becoming activated or repressed in concert; the global activation of many TE families in a genome has been observed, at least in genotypes with mutant epigenetic silencing pathways (Zemach *et al.* 2013; Panda *et al.* 2016). Second, the age profiles might represent the equilibrium state in which the rate of transposition and deletion of elements has been constant for some time.

We have shown that the number of vN and vS in an alignment of TE sequences from a single genome, declines as a function of the frequency of the variants in the alignment. This is consistent with the action of negative selection; elements that accumulate nonsynonymous mutations are less likely to transpose and hence have progeny, and hence have a high frequency in the alignment.

## Data availability

The list of genomes used to identify Sireviruses can be found in Supplementary Table 1. The Pfam HMM models used to determine the Open Reading Frame of each gene of the pol gene can be found in Supplementary S4. The code used to determine the vN/vS ratio can be found at https://github.com/AdamEyreWalker/Patterns_of_selection_in_the_evolution_of_a_transposable_element.

Supplemental material is available at *G3* online.

## Funding

This work was supported by Royal Society awards UF160222 and RGF/R1/180006.

## Conflicts of interest

None declared.

## Supplementary Material

jkac056_Supplemental_Material
