# Supplementary material for: Patterns of selection in the evolution of a transposable element
Source: G3 (Bethesda). 2022 Mar 9;12(5):jkac056. doi: 10.1093/g3journal/jkac056 (PMC9073684; doi:10.1093/g3journal/jkac056)
Supplement: jkac056_Supplemental_Material [file jkac056_supplemental_material.docx]

**Supplementary data:**

**Supplementary Figure S1.** Difference between a A) full-length Sirevirus LTR-retrotransposons and B) an intact element as defined in this work. The figures show the relative positions of some key features of a Sirevirus TE ; the primer binding site (PBS), the polypurine tract (PPT), the *gag* gene, and the *pol* gene, composed of 4 genes: the *protease* (PR), *integrase* (INT), *reverse transcriptase* (RT) and *ribonuclease-H* (RNaseH) genes. Note that some Sireviruses possess an envelope-like gene (Env) downstream the *gag-pol* domain.

**Supplementary Figure S2**. Method to retrieve the intact ORF for *gag* (A and B) and *pol* (C) in the *Opie* family. ORFs were first categorized as *gag* and *pol* based on their overlap with TE-specific HMM motifs (shown in Table S5). See Methods for more details about the pipeline. (A) shows the selected length (2325-2425nt) for the ORF of the *gag* gene (highlighted with the red bracket), (B) which were further selected and plotted to visualize their starting positions. The chosen starting positions (3; 24; 25; 40; 41) of the complete *gag* ORFs are highlighted with the red bracket. (C) In *pol*, we only select specific lengths (3234; 3237; 3246; 3249) and no starting position of the ORF, because the starting position within the internal domain can vary substantially within a family given individual insertions and deletions that elements may have acquired after integration. All *pol* ORFs contain the amino acid motif ADIFTK that is conserved among *Copia* LTR retrotransposons.

**Supplementary Table S1.** List of the genomes used for this analysis.

| Species | Website (accession date) | Publication (PubMed ID) |
| --- | --- | --- |
| *Zea mays* B73 | <http://ensembl.gramene.org/Zea_mays/Info/Index>  (June 2018) | 19965430 |
| *Musa acuminata* | <http://banana-genome-hub.southgreen.fr/download>  (May 2016) | 26984673 |
| *Glycine max* | <http://genome.jgi.doe.gov/pages/dynamicOrganismDownload.jsf?organism=Gmax> (May 2016) | 20075913 |
| *Asparagus officinalis* | <https://genome.jgi.doe.gov/portal/pages/dynamicOrganismDownload.jsf?organism=Aofficinalis> (June 2018) | 29093472 |
| *Helianthus annuus* | <https://genome.jgi.doe.gov/portal/pages/dynamicOrganismDownload.jsf?organism=Hannuus> (June 2018) | **28538728** |
| *Panicum hallii var. Hallii* | <https://genome.jgi.doe.gov/portal/pages/dynamicOrganismDownload.jsf?organism=PhalliiHAL> (June 2018) | 30523281 |
| Sorghum bicolor | <https://genome.jgi.doe.gov/portal/pages/dynamicOrganismDownload.jsf?organism=Sbicolor> (June 2018) | 29161754 |

**Supplementary table S2.** List of the Pfam HMM models used to determine the ORFs of each gene.

| Pfam ID | Description |
| --- | --- |
| PF14223 | *gag*-polypeptide of LTR copia-type |
| PF13961 | Domain of unknown function |
| PF00098 | Zinc finger |
| PF07727 | *Reverse transcriptase* (RNA-dependent DNA polymerase) |
| PF00665 | *Integrase* core domain |
| PF13976 | GAG-pre-*integrase* domain |

**Supplementary Table S3.** Codon sets used to count the numbers of synonymous and non-synonymous variants.

| Codon set | Amino acids | Number of codons | Codons |
| --- | --- | --- | --- |
| 1 | Ser/Pro/Thr/Ala | 16 | TCT, TCC, TCA, TCG, CCT, CCC, CCA, CCG, ACT,  ACC, ACA, ACG, GCT, GCC, GCA, GCG |
| 2 | Phe, Leu | 4 | TTT, TTC, CTT, CTC |
| 3 | Tyr/His | 4 | TAT, TAC, CAT, CAC |
| 4 | Lys/Glu | 4 | AAA, AAG, GAA, GAG |
| 5 | Cys/Arg | 4 | TGT, TGC, CGT, CGC |
| 6 | Arg/Gly | 4 | AGA, AGG, GGA, GGG |
| 7 | Iso/Val | 4 | ATT, ATC, GTT ,GTC |
| 8 | Asn/Asp | 4 | AAT, AAC, GAT, GAC |
| 9 | Ser/Gly | 4 | AGT, AGC, GGT, GGC |

**Supplementary Table S4.** Binomial test for each family, the number of NS and S sites for the genes were combined within each family.

| Family | Frequency category | Non-synonymous sites | Synonymous sites | p-value |
| --- | --- | --- | --- | --- |
| *Giepum* | 0 < x ≤ 1/64 | 215 | 454 | 0 |
|  | 1/64 < x ≤ 1/32 | 21 | 75 | 0 |
|  | 1/32 < x ≤ 1/16 | 7 | 57 | 0 |
|  | 1/16 < x ≤ 1/8 | 4 | 53 | 0 |
|  | 1/8 < x ≤ 1/4 | 3 | 30 | 0 |
|  | 1/4 < x ≤ 1/2 | 3 | 26 | 0 |
| *Hopie* | 0 < x ≤ 1/64 | 209 | 379 | 0 |
|  | 1/64 < x ≤ 1/32 | 13 | 47 | 0 |
|  | 1/32 < x ≤ 1/16 | 11 | 72 | 0 |
|  | 1/16 < x ≤ 1/8 | 6 | 38 | 0 |
|  | 1/8 < x ≤ 1/4 | 2 | 21 | 0 |
|  | 1/4 < x ≤ 1/2 | 5 | 25 | 0 |
| *Jienv* | 0 < x ≤ 1/64 | 0 | 0 | 1 |
|  | 1/64 < x ≤ 1/32 | 98 | 194 | 0 |
|  | 1/32 < x ≤ 1/16 | 20 | 58 | 0 |
|  | 1/16 < x ≤ 1/8 | 3 | 49 | 0 |
|  | 1/8 < x ≤ 1/4 | 6 | 37 | 0 |
|  | 1/4 < x ≤ 1/2 | 11 | 90 | 0 |
| *Opie* | 0 < x ≤ 1/64 | 324 | 518 | 0 |
|  | 1/64 < x ≤ 1/32 | 50 | 122 | 0 |
|  | 1/32 < x ≤ 1/16 | 40 | 121 | 0 |
|  | 1/16 < x ≤ 1/8 | 30 | 156 | 0 |
|  | 1/8 < x ≤ 1/4 | 27 | 141 | 0 |
|  | 1/4 < x ≤ 1/2 | 25 | 144 | 0 |
| *Ji* | 0 < x ≤ 1/64 | 372 | 609 | 0 |
|  | 1/64 < x ≤ 1/32 | 65 | 169 | 0 |
|  | 1/32 < x ≤ 1/16 | 36 | 145 | 0 |
|  | 1/16 < x ≤ 1/8 | 19 | 128 | 0 |
|  | 1/8 < x ≤ 1/4 | 29 | 123 | 0 |
|  | 1/4 < x ≤ 1/2 | 28 | 129 | 0 |
